# Supplementary material for: Reduced meiotic recombination in rhesus macaques and the origin of the human recombination landscape
Source: PLoS One. 2020 Aug 25;15(8):e0236285. doi: 10.1371/journal.pone.0236285 (PMC7447010; doi:10.1371/journal.pone.0236285)
Supplement: S1 File — (DOCX) [file pone.0236285.s001.docx]

Support information for “Reduced meiotic recombination in rhesus macaques and the origin of the human recombination landscape”

Cheng Xue^1^^,#a *^, Navin Rustagi^1^, Xiaoming Liu^2^, Muthuswamy Raveendran^1^,

R. Alan Harris^1^, Manjunath Gorentla Venkata^3^, Jeffrey Rogers^1^* and Fuli Yu^1^*

Table S1. List of samples and sample providers used in this study

| **Sample Name** | **Sample Source** | **Ancestry** | **Sex** |
| --- | --- | --- | --- |
| MMUL.IN-36468 | Caribbean Primate Research Center | Indian | F |
| MMUL.IN-18277 | Yerkes National Primate Research Center | Indian | F |
| MMUL.IN-19466 | Yerkes National Primate Research Center | Indian | M |
| MMUL.IN-24898 | Wisconsin National Primate Research Center | Indian | F |
| MMUL.IN-28499 | Wisconsin National Primate Research Center | Indian | M |
| MMUL.IN-28500 | Wisconsin National Primate Research Center | Indian | F |
| MMUL.IN-28507 | Wisconsin National Primate Research Center | Indian | M |
| MMUL.IN-28518 | Wisconsin National Primate Research Center | Indian | F |
| MMUL.IN-28535 | Wisconsin National Primate Research Center | Indian | M |
| MMUL.IN-28555 | Wisconsin National Primate Research Center | Indian | M |
| MMUL.IN-30119 | Wisconsin National Primate Research Center | Indian | F |
| MMUL.IN-30136 | Wisconsin National Primate Research Center | Indian | M |
| MMUL.IN-30158 | Wisconsin National Primate Research Center | Indian | M |
| MMUL.IN-30423 | Wisconsin National Primate Research Center | Indian | F |
| MMUL.IN-30424 | Wisconsin National Primate Research Center | Indian | M |
| MMUL.IN-31505 | Wisconsin National Primate Research Center | Indian | F |
| MMUL.IN-32510 | Wisconsin National Primate Research Center | Indian | F |
| MMUL.IN-32538 | Wisconsin National Primate Research Center | Indian | M |
| MMUL.IN-32754 | Wisconsin National Primate Research Center | Indian | F |
| MMUL.IN-33674 | Wisconsin National Primate Research Center | Indian | M |
| MMUL.IN-33707 | Wisconsin National Primate Research Center | Indian | M |
| MMUL.IN-34597 | New England Primate Research Center | Indian | F |
| MMUL.IN-34600 | New England Primate Research Center | Indian | M |
| MMUL.IN-34602 | New England Primate Research Center | Indian | M |
| MMUL.IN-34762 | Yerkes National Primate Research Center | Indian | F |
| MMUL.IN-34770 | Yerkes National Primate Research Center | Indian | F |
| MMUL.IN-35044 | Oregon National Primate Research Center | Indian | M |
| MMUL.IN-35045 | Oregon National Primate Research Center | Indian | M |
| MMUL.IN-35046 | Oregon National Primate Research Center | Indian | M |
| MMUL.IN-35048 | Oregon National Primate Research Center | Indian | M |
| MMUL.IN-35049 | Oregon National Primate Research Center | Indian | M |
| MMUL.IN-35051 | Oregon National Primate Research Center | Indian | F |
| MMUL.IN-35055 | Oregon National Primate Research Center | Indian | F |
| MMUL.IN-35059 | Oregon National Primate Research Center | Indian | M |
| MMUL.IN-35060 | Oregon National Primate Research Center | Indian | M |
| MMUL.IN-35061 | Oregon National Primate Research Center | Indian | M |
| MMUL.IN-35087 | California National Primate Research Center | Indian | F |
| MMUL.IN-35088 | California National Primate Research Center | Indian | M |
| MMUL.IN-35089 | California National Primate Research Center | Indian | F |
| MMUL.IN-35090 | California National Primate Research Center | Indian | M |
| MMUL.IN-35091 | California National Primate Research Center | Indian | F |
| MMUL.IN-35095 | California National Primate Research Center | Indian | M |
| MMUL.IN-35096 | California National Primate Research Center | Indian | F |
| MMUL.IN-35144 | New England Primate Research Center | Indian | M |
| MMUL.IN-35150 | New England Primate Research Center | Indian | M |
| MMUL.IN-35154 | New England Primate Research Center | Indian | M |
| MMUL.IN-35160 | New England Primate Research Center | Indian | M |
| MMUL.IN-35162 | New England Primate Research Center | Indian | M |
| MMUL.IN-35250 | New England Primate Research Center | Indian | F |
| MMUL.IN-35252 | New England Primate Research Center | Indian | F |
| MMUL.IN-35253 | New England Primate Research Center | Indian | F |
| MMUL.IN-35254 | New England Primate Research Center | Indian | M |
| MMUL.IN-35256 | New England Primate Research Center | Indian | F |
| MMUL.IN-35259 | New England Primate Research Center | Indian | F |
| MMUL.IN-35490 | Yerkes National Primate Research Center | Indian | F |
| MMUL.IN-35496 | Yerkes National Primate Research Center | Indian | F |
| MMUL.IN-35502 | Yerkes National Primate Research Center | Indian | F |
| MMUL.IN-35717 | Oregon National Primate Research Center | Indian | F |
| MMUL.IN-35718 | Oregon National Primate Research Center | Indian | F |
| MMUL.IN-35722 | Oregon National Primate Research Center | Indian | F |
| MMUL.IN-35724 | Oregon National Primate Research Center | Indian | F |
| MMUL.IN-35728 | Oregon National Primate Research Center | Indian | F |
| MMUL.IN-35730 | Oregon National Primate Research Center | Indian | F |
| MMUL.IN-35732 | Oregon National Primate Research Center | Indian | F |
| MMUL.IN-35864 | Tulane National Primate Research Center | Indian | M |
| MMUL.IN-35865 | Tulane National Primate Research Center | Indian | M |
| MMUL.IN-35866 | Tulane National Primate Research Center | Indian | F |
| MMUL.IN-35868 | Tulane National Primate Research Center | Indian | F |
| MMUL.IN-35871 | Tulane National Primate Research Center | Indian | M |
| MMUL.IN-35872 | Tulane National Primate Research Center | Indian | F |
| MMUL.IN-35873 | Tulane National Primate Research Center | Indian | M |
| MMUL.IN-35874 | Tulane National Primate Research Center | Indian | M |
| MMUL.IN-35875 | Tulane National Primate Research Center | Indian | F |
| MMUL.IN-35876 | Tulane National Primate Research Center | Indian | M |
| MMUL.IN-35883 | Tulane National Primate Research Center | Indian | M |
| MMUL.IN-35895 | Tulane National Primate Research Center | Indian | F |
| MMUL.IN-35902 | Tulane National Primate Research Center | Indian | M |
| MMUL.IN-35907 | Tulane National Primate Research Center | Indian | M |
| MMUL.IN-35916 | Tulane National Primate Research Center | Indian | F |
| MMUL.IN-35919 | Tulane National Primate Research Center | Indian | F |
| MMUL.IN-35921 | Tulane National Primate Research Center | Indian | F |
| MMUL.IN-35923 | Tulane National Primate Research Center | Indian | F |
| MMUL.IN-35957 | Tulane National Primate Research Center | Indian | F |
| MMUL.IN-35969 | Southwest National Primate Research Center | Indian | F |
| MMUL.IN-35972 | Southwest National Primate Research Center | Indian | M |
| MMUL.IN-35975 | Southwest National Primate Research Center | Indian | F |
| MMUL.IN-35976 | Southwest National Primate Research Center | Indian | F |
| MMUL.IN-35990 | Southwest National Primate Research Center | Indian | M |
| MMUL.IN-36332 | California National Primate Research Center | Indian | M |
| MMUL.IN-36355 | California National Primate Research Center | Indian | F |
| MMUL.IN-36357 | California National Primate Research Center | Indian | F |
| MMUL.IN-36359 | California National Primate Research Center | Indian | F |
| MMUL.IN-36371 | California National Primate Research Center | Indian | F |
| MMUL.IN-36374 | California National Primate Research Center | Indian | F |
| MMUL.IN-37730 | Wisconsin National Primate Research Center | Indian | M |
| MMUL.IN-37732 | Wisconsin National Primate Research Center | Indian | F |
| MMUL.IN-37733 | Wisconsin National Primate Research Center | Indian | F |
| MMUL.IN-37734 | Wisconsin National Primate Research Center | Indian | M |
| MMUL.IN-37735 | Wisconsin National Primate Research Center | Indian | M |
| MMUL.IN-37738 | Wisconsin National Primate Research Center | Indian | F |
| MMUL.IN-37739 | Wisconsin National Primate Research Center | Indian | M |
| MMUL.IN-37740 | Wisconsin National Primate Research Center | Indian | M |
| MMUL.IN-37741 | Wisconsin National Primate Research Center | Indian | M |
| MMUL.IN-37742 | Wisconsin National Primate Research Center | Indian | M |
| MMUL.IN-37745 | Wisconsin National Primate Research Center | Indian | F |
| MMUL.IN-37746 | Wisconsin National Primate Research Center | Indian | F |
| MMUL.IN-39345 | California National Primate Research Center | Indian | F |
| MMUL.IN-36460 | Caribbean Primate Research Center | Indian | F |
| MMUL.IN-36467 | Caribbean Primate Research Center | Indian | F |
| MMUL.IN-11414-01b | Wisconsin National Primate Research Center | Indian | F |
| MMUL.IN-11433-07b | Wisconsin National Primate Research Center | Indian | F |
| MMUL.IN-11433-08b | Wisconsin National Primate Research Center | Indian | M |
| MMUL.IN-36477 | Caribbean Primate Research Center | Indian | F |
| MMUL.IN-36476 | Caribbean Primate Research Center | Indian | F |
| MMUL.IN-36461 | Caribbean Primate Research Center | Indian | M |
| MMUL.IN-36462 | Caribbean Primate Research Center | Indian | M |
| MMUL.IN-36463 | Caribbean Primate Research Center | Indian | M |
| MMUL.IN-36475 | Caribbean Primate Research Center | Indian | M |
| MMUL.IN-36473 | Caribbean Primate Research Center | Indian | M |
| MMUL.IN-36474 | Caribbean Primate Research Center | Indian | M |
| MMUL.IN-36466 | Caribbean Primate Research Center | Indian | F |
| MMUL.IN-36471 | Caribbean Primate Research Center | Indian | F |
| MMUL.IN-36470 | Caribbean Primate Research Center | Indian | F |


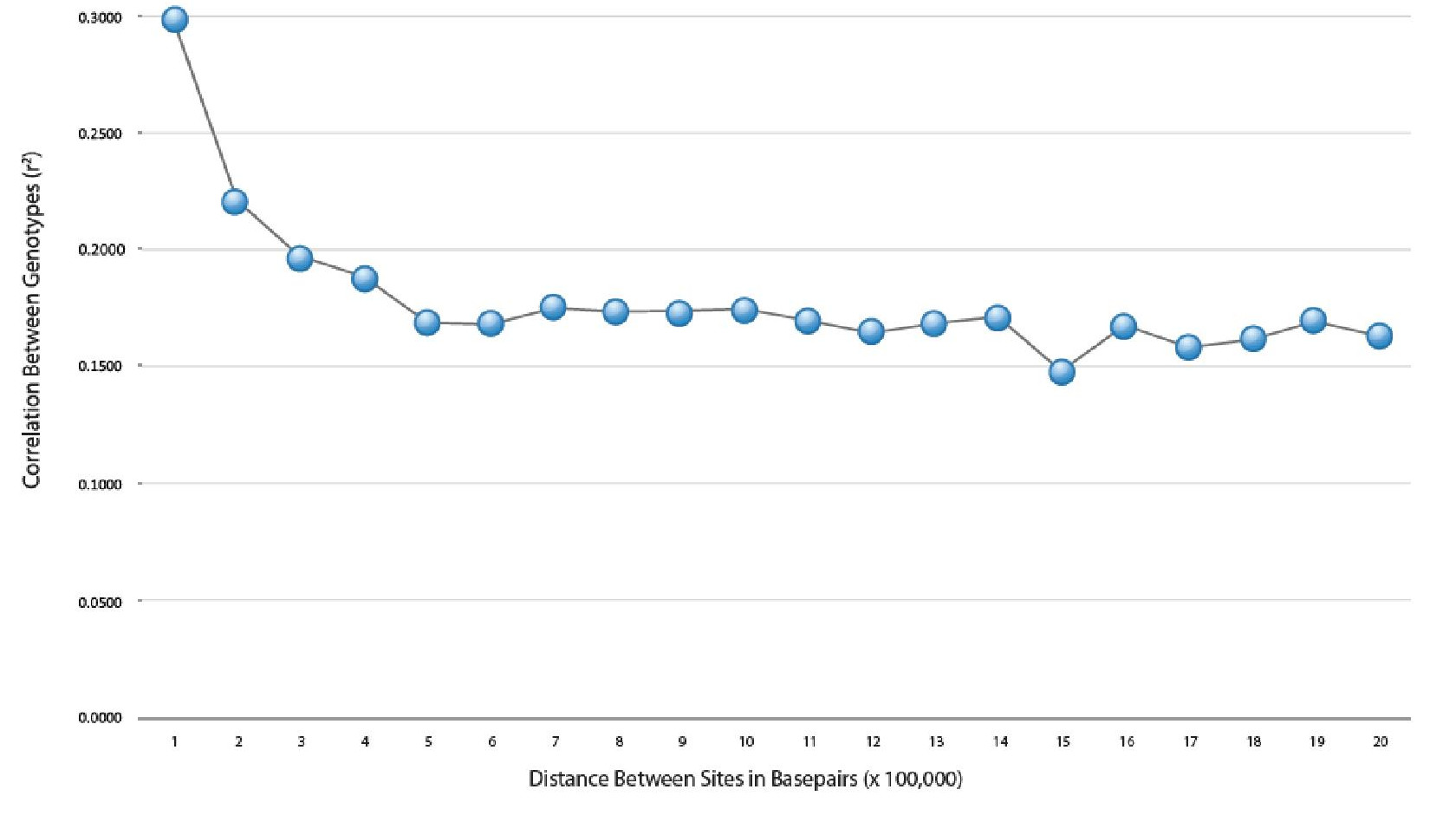


S1 Fig. Decay of linkage disequilibrium across chromosome 19 in rhesus genome. LD for each pair of SNPs was estimated by *plink*. The interval of window size is 100Kb and the correlation coefficients (r^2^) were calculated within each interval. S2 Fig. Correlation coefficients of recombination rates in syntenic regions between the rhesus and human genomes, calculated across a range of window sizes from 1 kb to 200 kb.


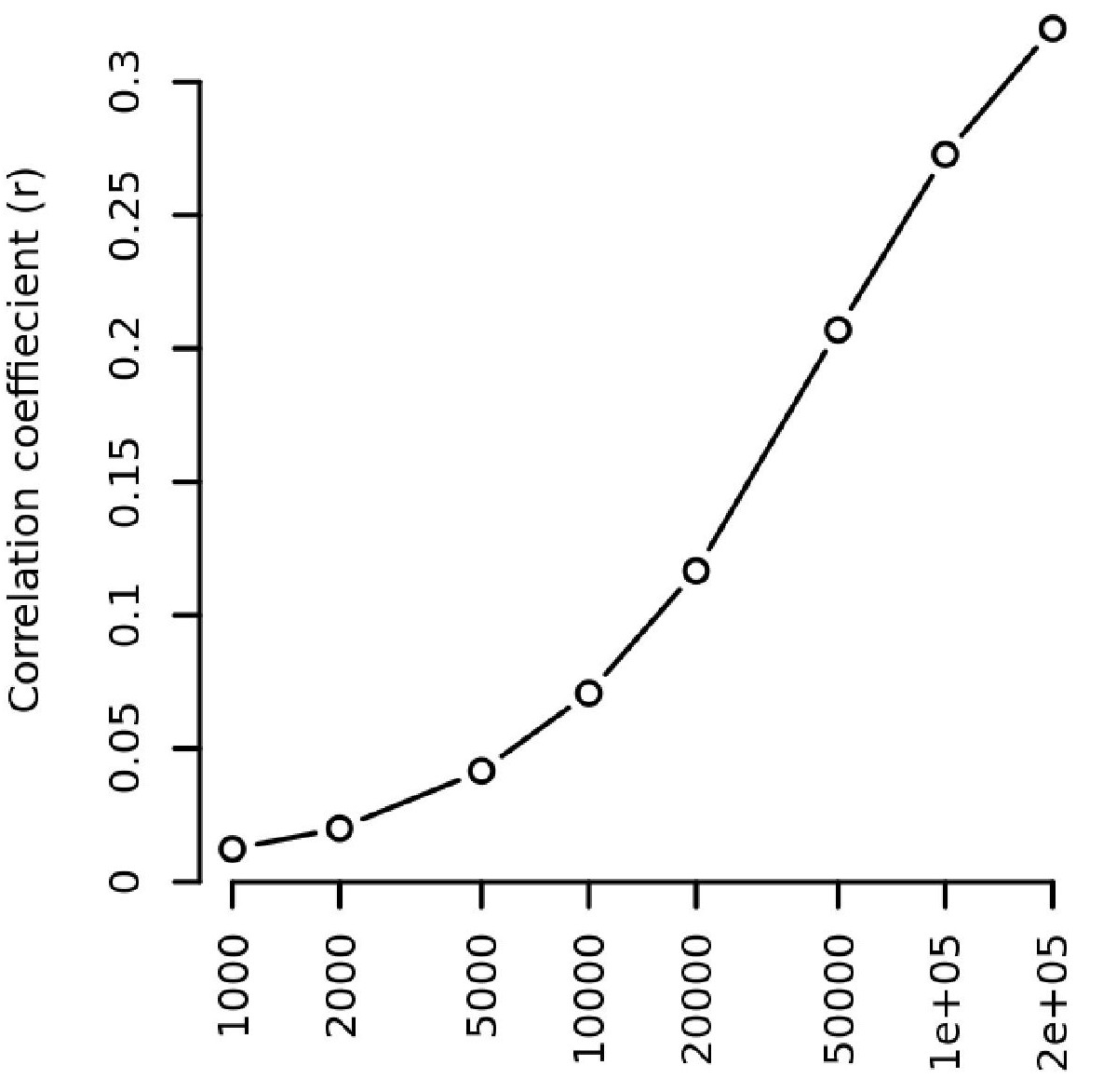


Bin size (bp)

S2 Fig. Correlation coefficients of recombination rates in syntenic regions between the rhesus and human genomes, calculated across a range of window sizes from 1 kb to 200 kb.
